# New application of dynamic magnetic resonance imaging for the assessment of deglutitive tongue movement

**DOI:** 10.1186/s40510-018-0245-x

**Published:** 2018-11-12

**Authors:** Issareeya Ekprachayakoon, Jun J. Miyamoto, Maristela Sayuri Inoue-Arai, Ei-ichi Honda, Jun-ichi Takada, Tohru Kurabayashi, Keiji Moriyama

**Affiliations:** 1 Maxillofacial Orthognathics, Graduate School of Medical and Dental Sciences, Tokyo Medical and Dental University (TMDU), 1-5-45 Yushima, Bunkyo-ku, Tokyo, 113-8549 Japan; 2 Chulalongkorn University-Tokyo Medical and Dental University Research and Education Collaboration Center, Tokyo Medical and Dental University (TMDU), 1-5-45 Yushima, Bunkyo-ku, Tokyo, 113-8549 Japan; 3 Oral and Maxillofacial Radiology, Tokushima University Graduate School, 3-17-15, Kuramoto-cho, Tokushima, 770-8504 Japan; 4 Oral and Maxillofacial Radiology, Graduate School of Medical and Dental Sciences, Tokyo Medical and Dental University (TMDU), 1-5-45 Yushima, Bunkyo-ku, Tokyo, 113-8549 Japan

**Keywords:** Cine-magnetic resonance (MR) imaging, Deglutition, Movement, Tongue

## Abstract

**Background:**

Deglutitive motion of the tongue may function to maintain tooth position. However, the causation between abnormal patterns of orofacial muscle function and dental malocclusion remains unclear. To clarify the pathogenic mechanism of malocclusion, it is important to determine the relative positional relationship between the tongue tip and incisor edge or the dorsal tongue and palate during deglutition. Here, we assessed the utility of 3-T segmented cine-magnetic resonance (MR) imaging, combined with static MR images for hard tissue visualization, in assessing the relationship between the tongue and the surrounding tissues during deglutitive tongue movement.

**Methods:**

Cine-MR images were acquired from three healthy female volunteers during deglutition who had no history of swallowing disorder or other chronic illness, normal alignment and occlusion, and a skeletal class I relationship. Three cine-MR images were taken during deglutition in accordance with an auditory cue for each volunteer. During static imaging, custom-made, contrast-medium-filled clear retainers were positioned in the mouth to allow visualization of the upper and lower incisors and hard palate boundaries. Static images were superimposed onto images of the three stages in deglutitive tongue movement, which were selected from a series of cine-MR images. These superimpositions were assessed five times by tracing cephalometric parameters to examine the reproducibility of the method.

**Results:**

Traces varied little across repeated measurements, and all subjects had a similar pattern of dorsal tongue movement. Tongue-to-palate contact increased slightly during the first to second stage of swallowing and abruptly increased during the second to third stage, while the tongue tip position remained constant.

**Conclusions:**

Segmented cine-MR imaging combined with static MR images is useful for assessing soft tissue motion during deglutition. This method is particularly useful in dentistry to evaluate the relationship between tongue function and maxillofacial morphology in terms of orthodontic treatment and orofacial myofunctional therapy, and for improving tongue movement during speech therapy.

**Electronic supplementary material:**

The online version of this article (10.1186/s40510-018-0245-x) contains supplementary material, which is available to authorized users.

## Background

Deglutition is one of the principal physiological functions of the oral cavity. Pressure from the tongue during deglutitive motion may contribute to the equilibrium that maintains tooth position [1]. It has long been debated whether abnormal patterns of orofacial muscle function, including tongue and lip postures, influence or create dental malocclusion, or conversely, whether malocclusion is the cause of abnormal tongue posture and function [2]. Therefore, deglutitive tongue function is of substantial interest not only to those in the dentistry field (orthodontists, dentists, and dental hygienists) but also to speech-language pathologists [3, 4] and other professionals working in the orofacial area.

Numerous techniques, such as cineradiography, videofluoroscopy, ultrasound scanning, and dynamic magnetic resonance imaging (MRI), can be used to assess dynamic tongue movement during deglutition [5]. MRI has the particular advantage of achieving excellent soft tissue resolution, without requiring exposure to radiation, and also allows for selection of the optimal image slice at the cross section of various planes. Currently, advanced cine-MRI allows for imaging of soft tissue motion with almost real-time resolution [6]. Although Foucart and colleagues [7] proposed the application of cine-MRI to study oropharyngeal structures, Ohkubo and colleagues [8] reported that the spatial resolution of the technique remains inadequate for the detection of some anatomical structures and indicated that it is necessary to use static MRI with high spatial resolution as reference points for cine-MRI. Yet, although MR images do not allow for the accurate visualization of hard tissue structures, Ng and colleagues [9] have reported distinguishing the incisor boundary on MRI during speech through the use of a customized retainer filled with ferric ammonium citrate (FAC)-containing contrast medium. However, no studies of tongue movement during deglutition with visualization of hard tissue structures, such as the anterior teeth or hard palate, have been reported to date.

High field-strength MR scanners have become increasingly available, and those with a 3-T field can achieve higher signal-to-noise ratios and thus provide better spatial resolution. This is particularly important in imaging of the head and neck, where high resolution is required to adequately identify the smaller anatomical structures [10]. Recently, real-time high-resolution MRI was used to study deglutition in normal subjects [11, 12]. These studies focused on treating dysphagia and on the temporal resolution of pharyngo-oral movement, such as the timing of velopharyngeal closure, glottal closure, and esophageal opening and closure; this analysis did not spatially resolve the movement of these anatomical structures.

Protrusion of the tongue between the upper and lower incisors or cuspids, with no molar contact during swallowing (defined as infantile swallowing), is strongly associated with an anterior open bite [13]. Therefore, to clarify the pathogenic mechanism of malocclusion, it is important to assess positional changes of the tongue caused by its protrusion and elevation and to understand the relative positional relationship between the tongue tip and incisor edge during normal deglutition. The aim of this study was thus to evaluate the ability of 3-T segmented cine-MRI to assess deglutitive tongue movement when used in conjunction with a static MR image in normal subjects.

## Methods

This study was conducted after institutional approval from the Clinical Investigation Ethics Committee of Tokyo Medical and Dental University, Japan (reference number 1025). Before the experiment, the subjects were informed in detail about the nature of the experiment and gave their written informed consent to participate in the study. Three healthy female volunteers (ages 27.5, 29.0, and 30.5 years) with no history of swallowing disorder or other chronic illness were enrolled. All subjects had normal dental alignment and occlusion with a skeletal class I relationship.

**Fig. 1:**
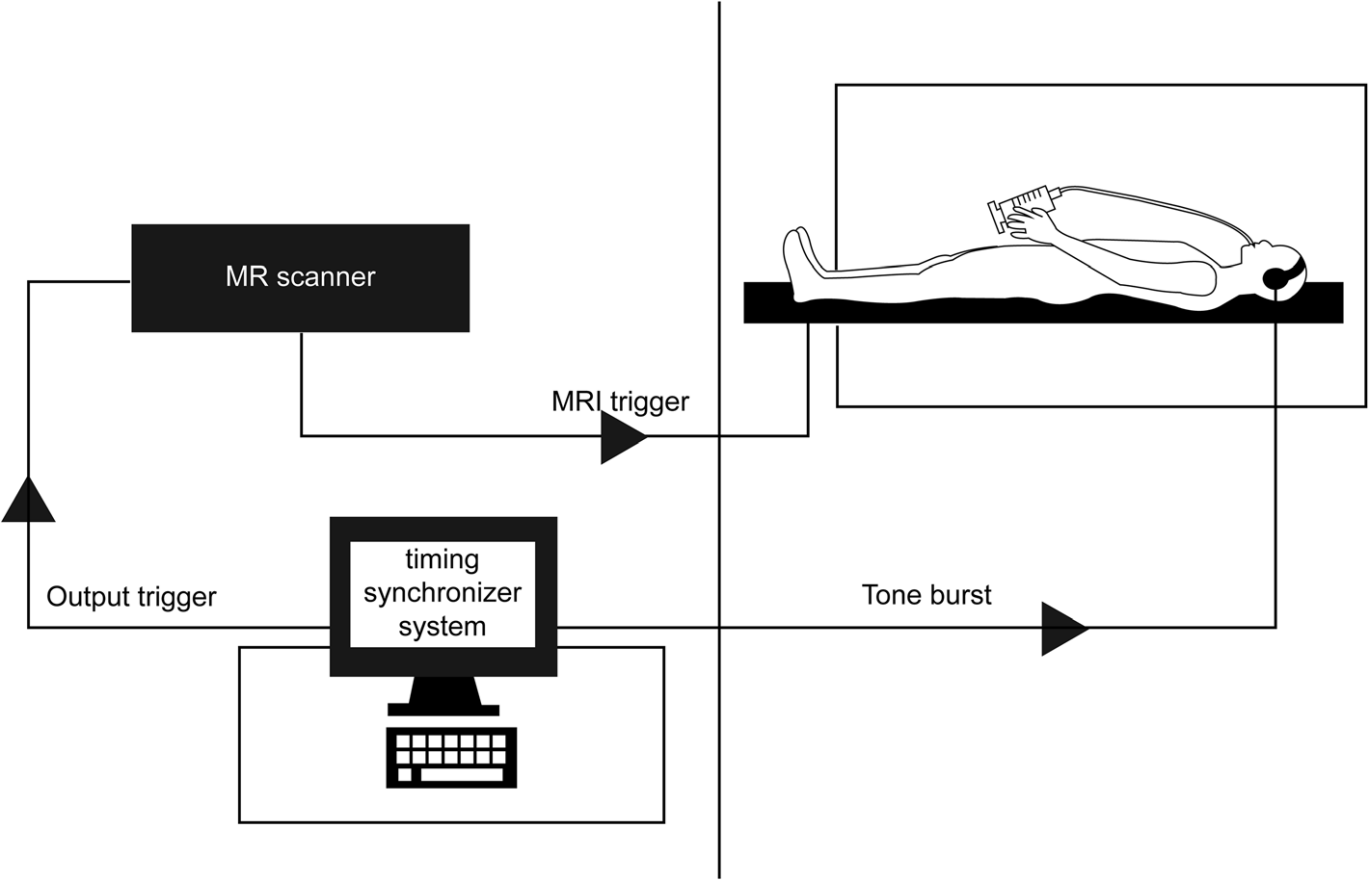
Experimental settings for cine-magnetic resonance imaging (MRI)

A 3-T MR scanner (Magnetom Spectra; Siemens AG, Erlangen, Germany) with a head and neck coil was used and connected to custom-made circuitry, with an input terminal for an external trigger pulse to control the timing of the scanning sequence (Fig. 1).

First, three plastic tube landmarks filled with contrast medium, consisting of FAC and sodium bicarbonate in a gel form, were positioned along the subject’s facial midline at the forehead, nasal tip, and chin and secured with tape. To capture the dynamic images, subjects lay supine in the scanner with no appliances in their mouths; headphones were used to stabilize the head within the coil and to provide an auditory cue to synchronize deglutition with scanning. Water (5 mL) was provided via a syringe and tubing to aid swallowing. Midsagittal plane images were acquired, each with a 256 mm × 256 mm field-of-view, a pixel size of 1 mm × 2 mm, and a slice thickness of 4 mm. Each image run was acquired using a gradient echo (GRE) sequence for a cardiac cine (repetition time [TR] = 90 ms; echo time [TE] = 2.07 ms; flip angle = 12°). During data acquisition, an external trigger pulse was fed to the MR scanner and deglutition events were performed six times with auditory stimuli, which were synchronized with the trigger pulse. This scanning during six deglutition events formed one series of sequential dynamic images consisting of more than 100 images. The time required for data acquisition was approximately 4 min. The dynamic images were recorded for three cycles to measure intra-individual variability.

**Fig. 2:**
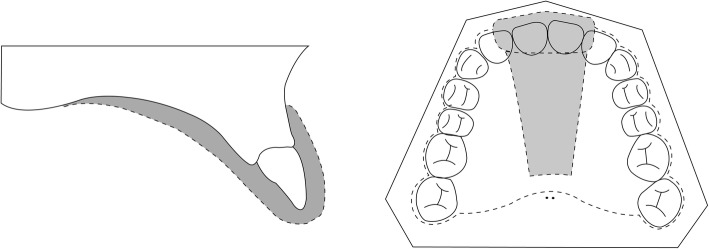
Schematic illustration of a customized clear BIOSTAR plate. The plate has space around the central incisors and along the middle of the hard palate. The dotted line represents the boundary of the retainer. The gray-colored area represents the space filled with contrast medium gel

**Fig. 3:**
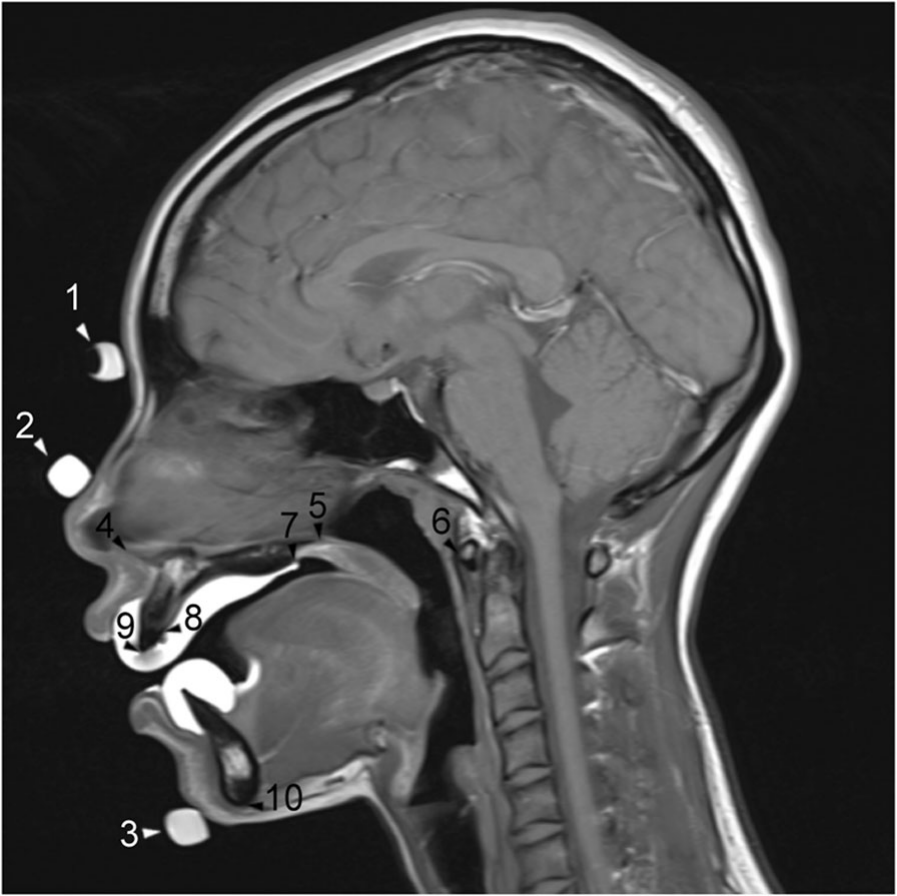
A representative static magnetic resonance image. Landmarks on the (1) forehead, (2) nasal tip, (3) chin, (4) ANS (anterior nasal spine), (5) PNS (posterior nasal spine), (6) C1 (the most anterior point of the atlas), (7) PM (point at which the soft and hard palates intersect), (8) AM (boundary point between the maxillary central incisor and the palatal mucosa), (9) I (point at the edge of the maxillary incisor), and (10) Me (the most posterior point on the mandibular symphysis). For abbreviations, see Table 1

After capturing the dynamic images, subjects were then asked to insert customized clear BIOSTAR (3171 Imprelon S; SCHEU-Dental Technology, Iserlohn, Germany) retainers to the upper and lower dental arches, which were made on dental casts before the experiment. These retainers, with space around the central incisors and the middle portion of the hard palate (Fig. 2), were filled with contrast medium and placed into the subjects’ mouths to visualize the central incisors and the hard palate. A static T1-weighted image was obtained using a turbo spin echo (TSE) sequence (TR = 500 ms; TE = 20 ms). Each image had a 256 mm × 256 mm field-of-view, a pixel size of 0.5 mm × 0.5 mm, and a slice thickness of 4 mm. The height and width of the pixels for images acquired by GRE and TSE sequences were set to an exact ratio of 2:1 or 4:1 to eliminate error from linear interpolation during superimposition. A representative static image is shown in Fig. 3.

**Fig. 4:**
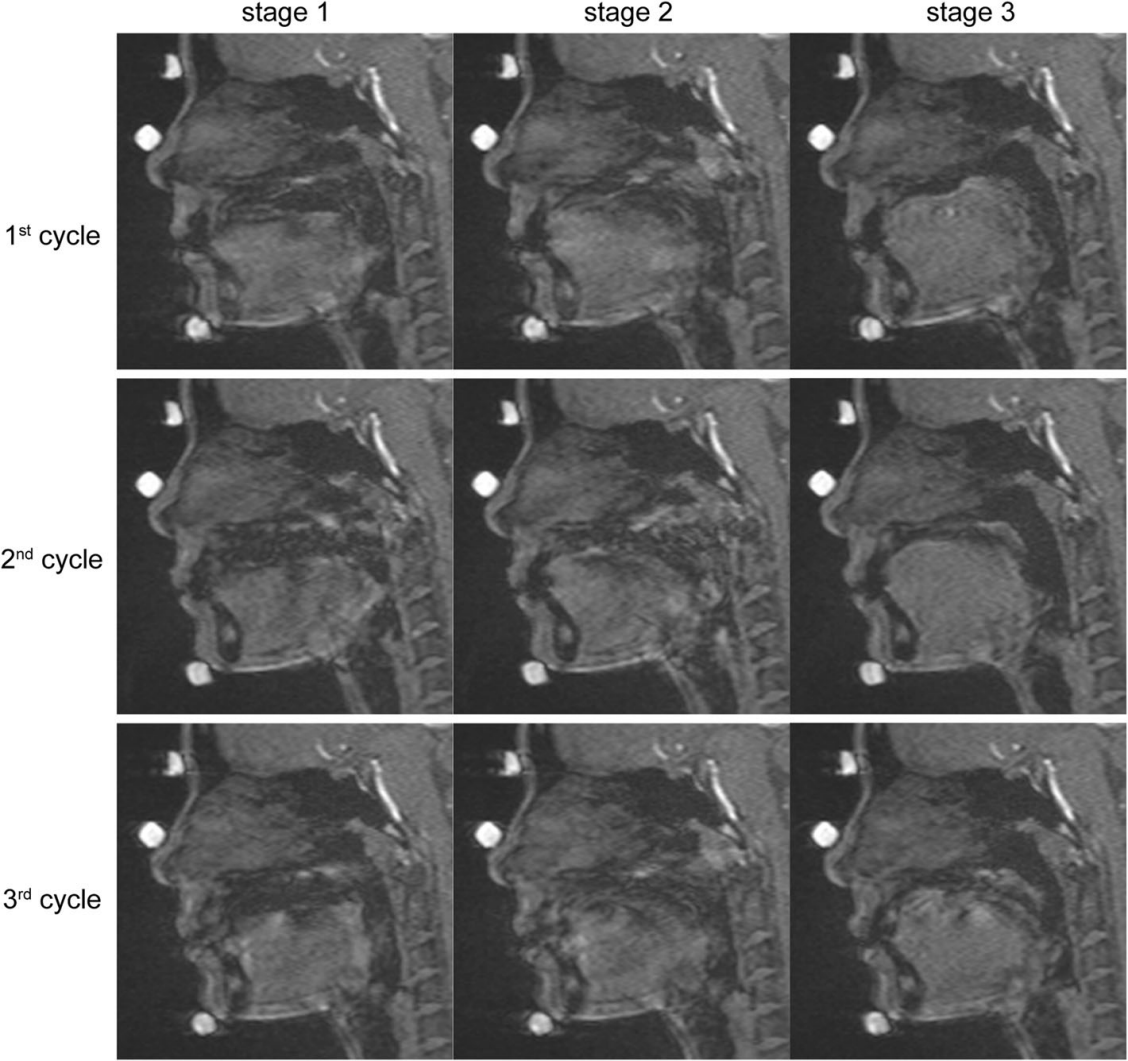
Acquired images of three cycles of stage 1, stage 2, and stage 3 deglutition. Three cycles were taken at 0 ms, 90 ms, and 270 ms, respectively

All acquired images were transferred to a computer with a proprietary image-capturing program. From a series of dynamic images, we selected images that corresponded with the three stages of deglutition established by Fujiki and colleagues [14]: stage 1, loss of contact of the dorsal tongue with the soft palate; stage 2, passage of the bolus head across the posterior/inferior margin of the ramus of the mandible; and stage 3, passage of the bolus head through the opening of the esophagus. A representative image of each stage is shown in Fig. 4.

**Fig. 5:**
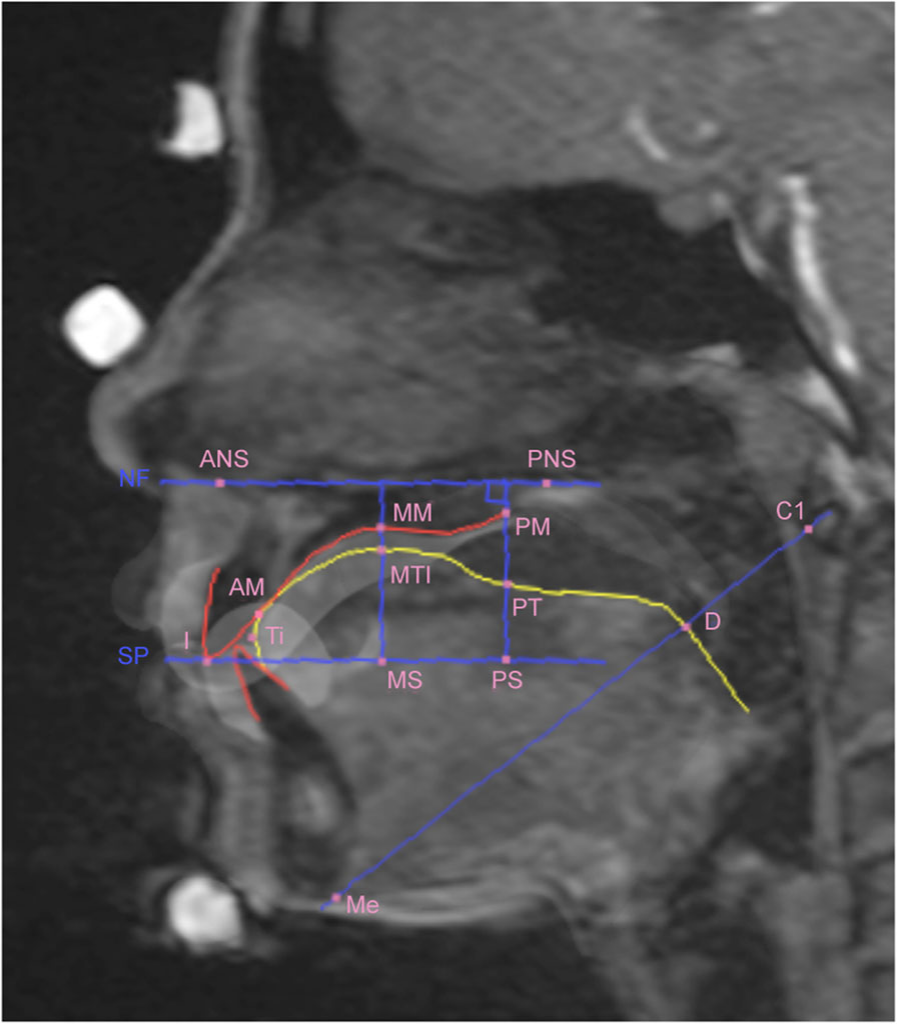
Lines and points on the cine-magnetic resonance image after superimposition of the static image. Red line: tracing of the upper incisor, hard palate, and lower incisor. Yellow line: tracing of the tongue. Blue line: the planes used in the analysis. Pink dots: the points used in the analysis. Please refer to Table 1 for abbreviations

Superimposition of the static MR and selected dynamic images was undertaken using Adobe Photoshop CS6 (Adobe Systems, Inc., San Jose, CA, USA). For these superimpositions, custom-made landmarks over the forehead, nasal tip, and chin were used. Anatomical structures were traced after superimposition, particularly the boundaries of the upper incisor, lower incisor, and hard palate, using the image of the clear retainers. Cephalometric reference points were then traced on the superimposed images (Fig. 5).

**Fig. 6:**
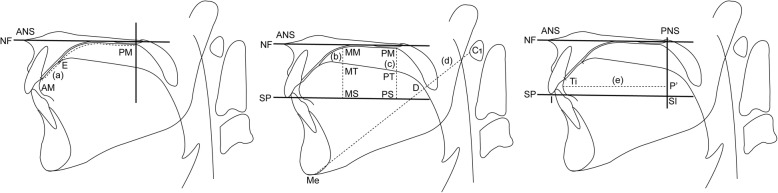
Linear measurements. (a) Contact of the tongue and palate, AM-E/AM-PM. (b) Front part of the dorsal tongue, MM-MT/MM-MS. (c) Mid part of the dorsal tongue, PM-PT/PM-PS. (d) Rear part of the dorsal tongue, C1-D/C1-Me. (e) Tongue tip, P′-Ti/SI-I. MM-MT, MM-MS, PM-PT, PM-PS, C1-D, C1-Me, and SI-I are distances measured in a straight line. P′-Ti is the shortest distance from a line perpendicular to the palatal plane (through the PNS) to Ti (modified from Fujiki et al. [14]). For abbreviations, see Table 1

Linear measurements for determining the extent of contact between the palate and tongue and the position of the dorsal tongue and palate modified from the cineradiographic study of Fujiki and colleagues [14] were taken for each stage of deglutition. Parameters used in the analysis are described in Table 1 and Fig. 6. All parameters were calculated as ratios expressing the tongue position in the oral cavity. The ratio of the contact area of the tongue and palate (Fig. 6a) indicates that the more the ratio increases, the more the contact area increases. The ratios of the superior space of the oral cavity proper at the front (Fig. 6b), middle (Fig. 6c), and rear (Fig. 6d) sections of the dorsal tongue indicate that the more the ratio increases, the more the space increases, indicating that the tongue moves further downward. The ratio of the tongue tip position to the incisal edge position (Fig. 6e) indicates that the more the ratio increases, the more the tongue tip position moves anteriorly. The reproducibility of the method was examined by measuring the selected images of each of the three cycles of deglutition of the representative subject five times by a single investigator (IE), and intra-subject variability between the three cycles of deglutition was quantified by the measurements between the three cycles. The inter-subject variability was confirmed by averaging each of the three stages of all cycles within each subject.

**Table 1:** Reference points and planes (modified from Fujiki et al. [14])

Landmark	Definition
ANS	Point at the tip of the anterior nasal spine
PNS	Point at the dorsal limit of the maxilla
Me	The most posterior point on the mandibular symphysis
I	Point at the edge of the maxillary incisor
C1	The most anterior point of the atlas
NF	Plane through ANS and PNS
SP	Plane passing the edge of the maxillary incisor and parallel to the palatal plane
AM	Boundary point between the maxillary central incisor and the palatal mucosa
E	Point nearest to the tongue base in the region of contact between tongue and palatal mucosa
MM	Point at which a line perpendicular to NF drawn through a point equidistant between ANS and PNS intersects the palatal mucosa
MT	Point at which a line perpendicular to NF drawn through a point equidistant between ANS and PNS intersects the dorsal tongue
MS	Point at which a line perpendicular to NF drawn through a point equidistant between ANS and PNS intersects SP
PM	Point at which the soft and hard palates intersect
PT	Point at which a line perpendicular to NF through point PM intersects the dorsal tongue
PS	Point at which a line perpendicular to NF through point PM intersects SP
D	Point at which a line through Me and C1 intersects the dorsal tongue
Ti	Tongue tip
SI	Point at which a line perpendicular to NF through PNS intersects SP

## Results

A series of midsagittal plane images extracted from a dynamic series acquired on an MR scanner (3-T field strength) is shown for a representative subject in Additional file 1: Movie S1. The images are shown for the subject at rest, in the starting position, and during swallowing.

**Fig. 7:**
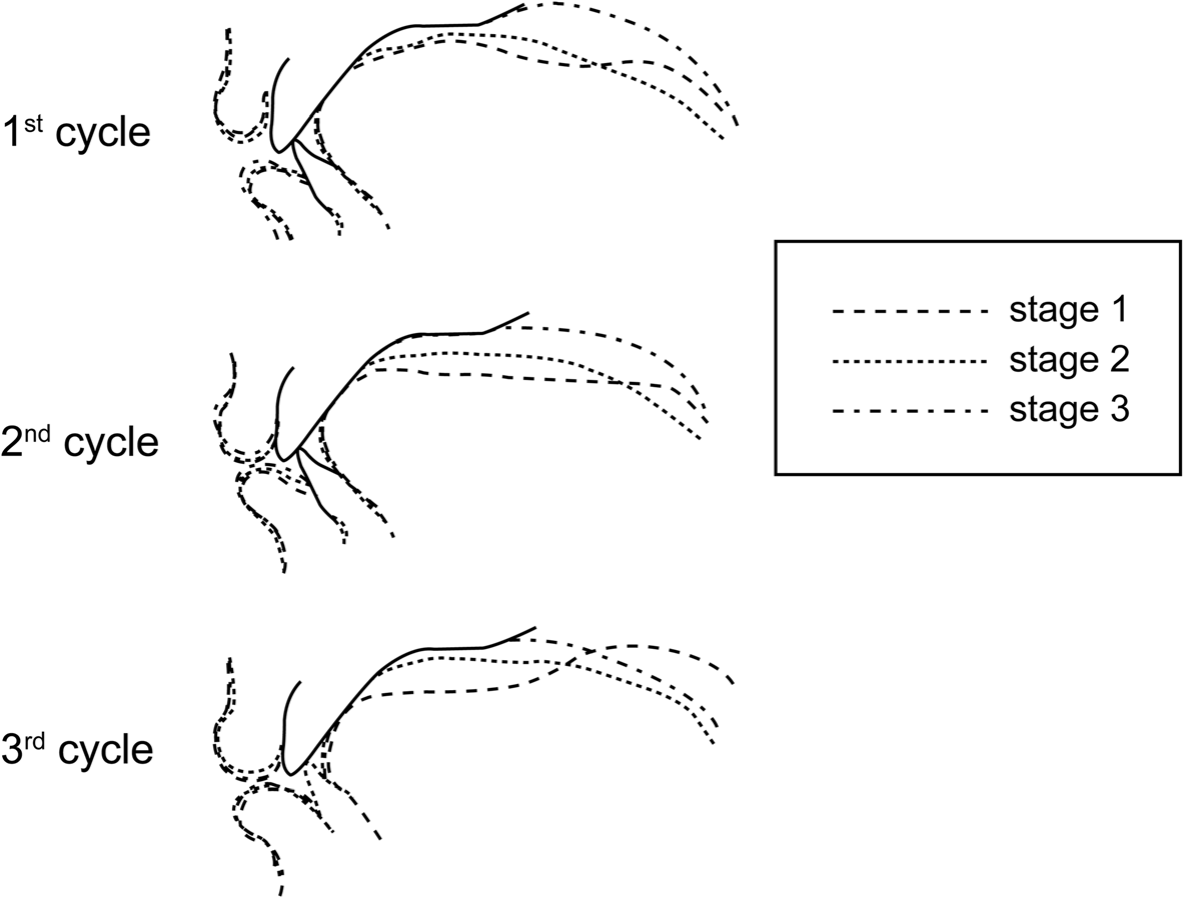
Comparison of images from a representative subject at each stage of deglutition. Tracings were made at stages 1, 2, and 3 of deglutition for each cycle

**Fig. 8:**
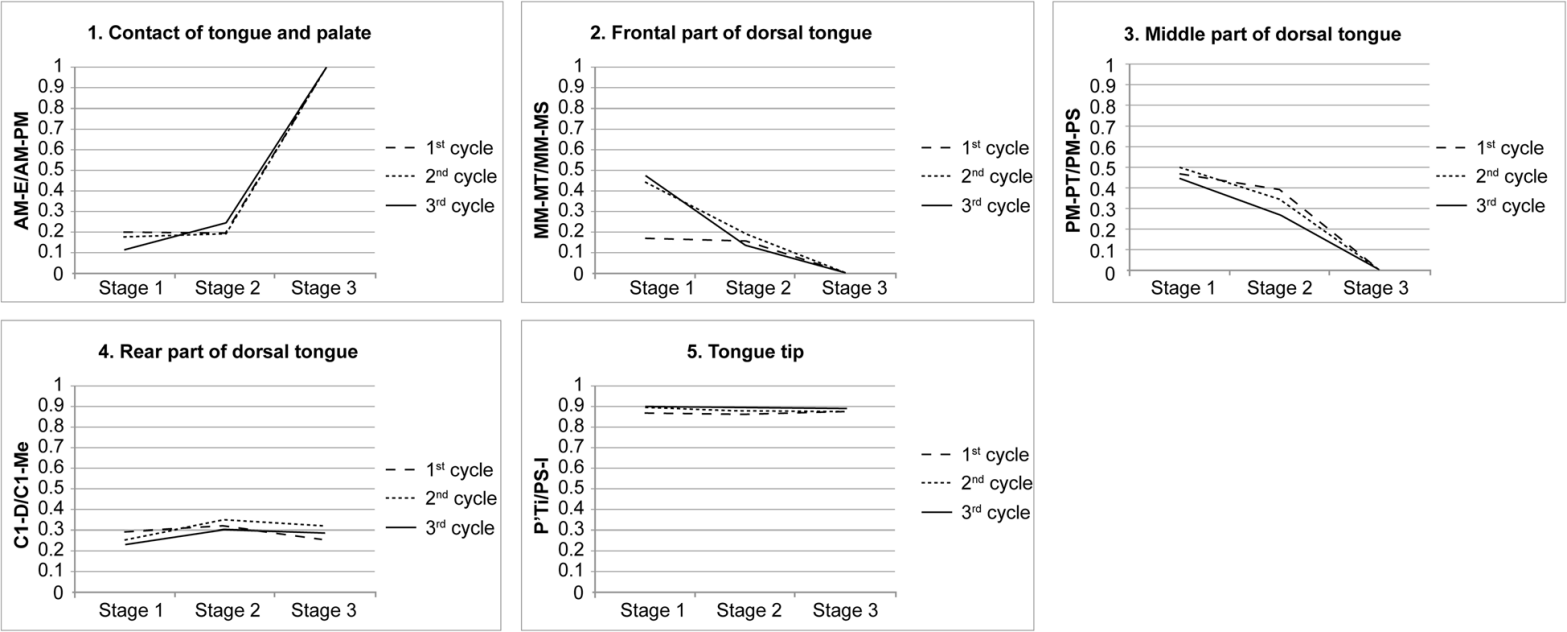
Comparison of linear measurements recorded in each cycle of deglutition in a representative subject. For abbreviations, see Table 1. Linear measurements are as described in Fig. 6

**Fig. 9:**
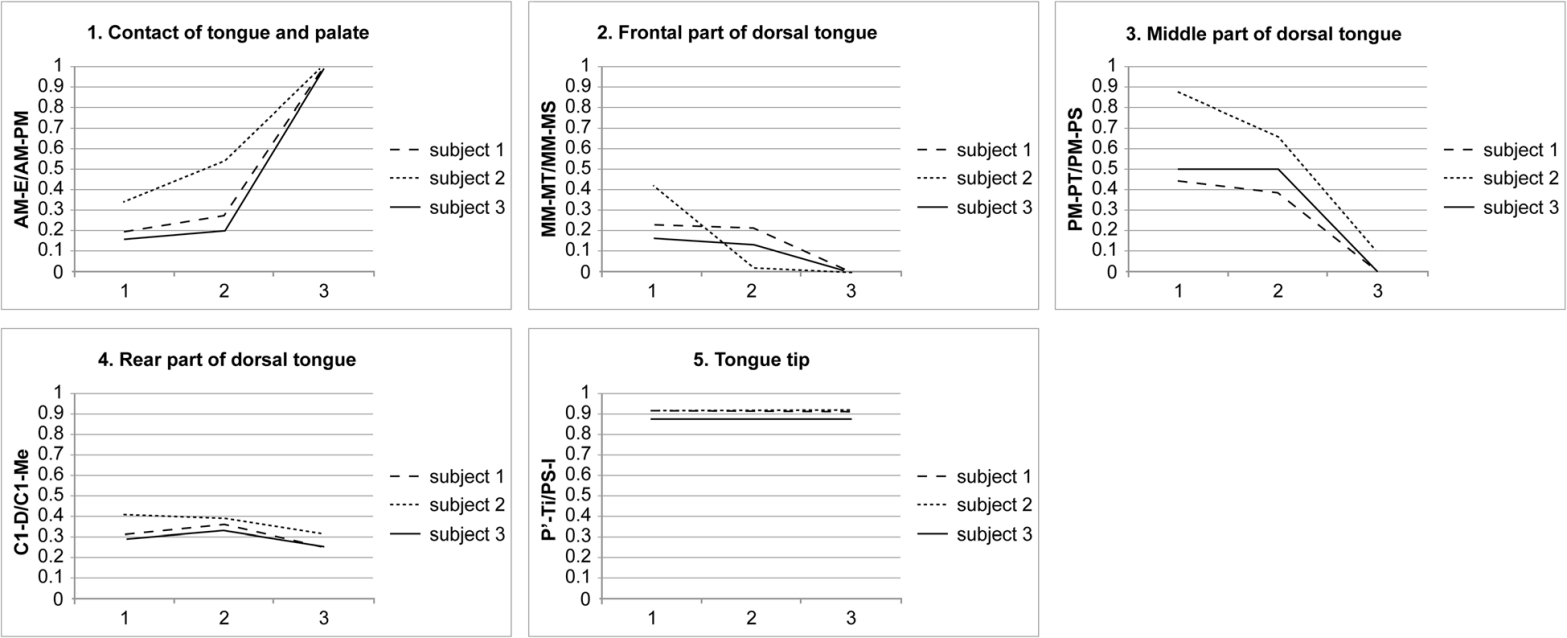
Comparison of linear measurements between the three subjects. For abbreviations, see Table 1. Linear measurements are as per those described in Fig. 6)

The descriptive data with standard deviations to examine the reproducibility of this method are shown in Table 2. The results of intra-subject variability between the three cycles of deglutition, and superimposition of sequential tracings of the lip, teeth, and tongue of the representative subject are illustrated in Fig. 7, and a comparison of linear measurements taken during each cycle of deglutition of the same subject is shown in Fig. 8. From the results of the inter-subject variability (Fig. 9), the three subjects showed broadly similar swallowing patterns.

**Table 2:** Measurements (ratio) for each stage of deglutition (*n* = 5 per subject)

	Mean	SD
Contact between tongue and palate (AM-E/AM-PM)		
Stage 1	0.17	0.05
Stage 2	0.21	0.03
Stage 3	1.00	0
Front part of dorsal tongue (MM-MT/MM-MS)		
Stage 1	0.36	0.17
Stage 2	0.16	0.03
Stage 3	0	0
Middle part of dorsal tongue (PM-MT/PM-MS)		
Stage 1	0.47	0.03
Stage 2	0.33	0.06
Stage 3	0	0
Rear part of dorsal tongue (C1-D/C1-Me)		
Stage 1	0.26	0.03
Stage 2	0.32	0.02
Stage 3	0.29	0.03
Tongue tip (P′-Ti/PS-I)		
Stage 1	0.88	0.02
Stage 2	0.88	0.02
Stage 3	0.88	0.01

For definitions of abbreviations, please see Table 1

Beginning at the time that the bolus moved from stage 1 to stage 2, as it crossed the ramus, there was a slight increase in the contact between the tongue and the palate (Figs. 8 and 9). At this time, there was no significant movement of the frontal or middle parts of the dorsal tongue in subjects 1 or 3; however, movement at both parts was abruptly elevated in subject 2 (Fig. 9). Subsequently, from stage 2 to stage 3, by the time the bolus had passed through the opening of the esophagus, contact between the tongue and palate was sharply increased (Figs. 8 and 9). The frontal, middle, and rear parts of the tongue were elevated, and the elevation was particularly notable in the middle part (Figs. 8 and 9). There was no difference in the position of the tongue tip at any stage of deglutition (Figs. 8 and 9).

## Discussion

We found that segmented cine-MRI offers several advantages over other techniques in terms of spatial resolution for monitoring tongue movements. We obtained high-quality images and a detailed depiction of the anatomical structures during swallowing with a 3-T scanner and a small pixel size. By segmenting MRI signals and filling those into each k-space, the TR can be shortened and the apparent temporal resolution markedly increased [15]. Using this method, cine-MR images allowed for the fine temporal evaluation of swallowing movements. Moreover, this method also placed fewer demands on subjects, because fewer deglutition events were required to monitor one cycle of deglutition.

Superimposition of static images also allowed for better visualization of those structures barely detectable on cine images alone, including the bony structures of the nasal spines, menton, and atlas, which made the analysis more precise. Consequently, we found that the point measured at the dorsal limit of the maxilla (PNS) was sometimes positioned beyond the soft palate. In the original cephalometric study of Fujiki and colleagues [14], the position of the middle part of the dorsal tongue was determined by the distance from the dorsal tongue surface to the palatal mucosal surface, at the line crossing perpendicular to the plane, through the tip of the anterior nasal spine (ANS), and the dorsal limit of the maxilla (NF, the plane through the ANS and PNS; see Table 1 and Fig. 6). Nevertheless, as the soft palate moves during swallowing, determining the position of the tongue using this line could be inaccurate. Therefore, we moved the original parameter to the junction between the hard and soft palates (PM in Fig. 3). The use of a customized retainer containing contrast medium during the acquisition of static images allowed visualization of the hard palate boundary, as well as the incisors, thus allowing the relationship between the tongue and teeth or tongue and hard palate to be understood more completely. Indeed, in a previous study using real-time balanced turbo field echo cine-MRI with a 1.5-T MR scanner to evaluate tongue movements in subjects with anterior dental open bites during deglutition [16], the authors did not clarify the boundaries of the anterior teeth and the hard palate, which makes it difficult to detect accurately the positions of anatomical structures, although they also used the same measurement of the length as defined by Fujiki et al. [14]. Additionally, the reproducibility of our current technique is emphasized by the small standard deviation in repeated measurements, as compared with that in the previous study [16], even though our number of subjects was much smaller.

To compare between the current and previous methods for taking dynamic images during deglutition with a 3-T MR scanner, a clinical machine without special customization can be used; however, there are also other advantages of this method. The previously reported method using a radial fast low-angle shot (FLASH) sequence with a short TR—differing from a segmented cine-MR scan, which has a higher temporal resolution (41.23 ms) [11]—is not installed on commercially available MR scanners. This method requires multiple lines for filling k-space in a TR. Most commercially available MRI systems fill the k-space line-by-line when using radial k-space filling, whereas the FLASH sequence seems to be customized to allow for the use of a shorter TR. When the number of lines required to fill k-space in a TR is the same, the TR in Cartesian sampling is shorter than that in radial sampling. Meanwhile, radial sampling has disadvantages: because the radial k-space filling causes contrast reduction in small anatomical structures [17] and has a low signal-to-noise ratio [18], it is necessary to increase the slice thickness and pixel size and/or use a surface coil [11] to improve contrast. We used segmented k-space cine mode with Cartesian sampling in our experiments for the following reasons: First, the temporal resolution of normal radial sampling is decreased compared with Cartesian sampling because of the longer TR. Second, the contrast between the soft tissues decreases because of a decrease in the signal intensity. Third, there is resultant decrease in spatial resolution, which makes it difficult to diagnose the anatomical parts in detail.

Deglutition consists of three stages: the oral phase, during which the bolus is propelled from the oral cavity into the pharynx by voluntary movements of the tongue; the pharyngeal phase, which starts with triggering of the swallowing reflex and ends with the closure of the upper pharyngeal sphincter; and the esophageal phase, in which the bolus is transported to the stomach [19, 20]. The stages we analyzed represent the end of the oral phase of swallowing (stage 1), the beginning of the pharyngeal phase (stage 2), and the end of the pharyngeal phase (stage 3). We propose that the variability in our recordings in stages 1 and 2 might be a consequence of voluntary movement by the subjects. We also found that there was no difference in the position of the tongue tip at any stage. A previous study [14] reported that the position of the tongue tip in patients with an anterior open bite was significantly more anterior than that of individuals with normal occlusion, in all stages of swallowing.

Although there are some general disadvantages of the segmented cine-MRI, the following points do not hinder its usefulness. First, to improve temporal resolution while maintaining spatial resolution, it is necessary to increase the number of repetitions of deglutition events. When using cine-MRI, however, this repetition may be acceptable because few deglutition events were used. Second, blurring and distortion of images in segmented cine-MRI were observed when a rhythmical movement was not achieved because the images are created by combining k-space data at different time points rather than by continuous scanning. Conversely, evaluation of the blurring and distortion of the images may be useful for diagnosing deglutition disorders, such as those involving dysfunction of rhythmical deglutition.

The study had several limitations. First, subjects had to perform deglutition in a supine position, which may be considered unnatural, albeit previous deglutition studies have also been performed with subjects in a supine position [11, 21]. Because this posture might have affected the exact time course of deglutition, further studies to capture images in a sitting position should be conducted [22]. Finally, the dynamic images often become invisible or distorted and linear measurements may be incorrect because metallic appliances in the oral cavity, such as orthodontic appliances, produce a range of artifacts depending on the location and type of metal [23]. The authors concluded, however, that orthodontists should not necessarily remove all metallic appliances before MRI examination and that caution should be exercised when the distorted image is measured. Therefore, to compensate for this limitation, it is necessary to understand the properties of measurement errors caused by metallic artifacts in the dynamic images.

## Conclusion

We found that 3-T segmented cine-MRI, used in conjunction with static MRI, is as useful as the low intra-subject variation method as a means of assessing soft tissue motion with the visualization of hard tissue structures, including the teeth, during deglutition. The technique used in the current study is useful for evaluating the relationship between tongue function and maxillofacial morphology, in terms of orthodontic treatment and orofacial myofunctional therapy, and for assessing unusual tongue motion, which causes swallowing abnormalities and improper posture of the tongue. Furthermore, it also allows tongue movements to be evaluated after surgery for tongue cancer, after brain infarction, during speech therapy after partial tongue resection, or during speech therapy for uraniscolalia.

## Additional file

Additional file 1: **Movie S1.** A representative movie taken during deglutition. (MP4 1920 kb)

## Abbreviations

FAC: Ferric ammonium citrate
FLASH: Fast low-angle shot
GRE: Gradient echo
MRI: Magnetic resonance imaging
TR: Repetition time
TSE: Turbo spin echo
